# Stability Comparison of Recordable Optical Discs—A Study of Error Rates in Harsh Conditions

**DOI:** 10.6028/jres.109.038

**Published:** 2004-10-01

**Authors:** Oliver Slattery, Richang Lu, Jian Zheng, Fred Byers, Xiao Tang

**Affiliations:** National Institute of Standards and Technology, Gaithersburg, MD 20899-8951

**Keywords:** archiving, CD-R, digital preservation, DVD-R, error rates, life expectancy

## Abstract

The reliability and longevity of any storage medium is a key issue for archivists and preservationists as well as for the creators of important information. This is particularly true in the case of digital media such as DVD and CD where a sufficient number of errors may render the disc unreadable. This paper describes an initial stability study of commercially available recordable DVD and CD media using accelerated aging tests under conditions of increased temperature and humidity. The effect of prolonged exposure to direct light is also investigated and shown to have an effect on the error rates of the media. Initial results show that high quality optical media have very stable characteristics and may be suitable for long-term storage applications. However, results also indicate that significant differences exist in the stability of recordable optical media from different manufacturers.

## 1. Introduction

Recordable optical disc media contains an organic dye layer whose transparency can be altered either to absorb a laser beam or to allow the beam to pass through to a reflective layer behind the dye [1,2]. The nature of this organic dye is such that when the internal energies of its molecules reach a particular threshold, an irreversible chemical reaction occurs, and the dye layer loses its transparency. This property allows a high-energy beam to “write” data by burning “pits,” in the form of dark marks, to the disc during recording. A low powered laser reads the data by either passing through the transparent dye layer (without causing any molecular change) to the reflective layer or by being absorbed by the nontransparent marks in the dye.

Due to the organic nature of the dye, degradation and breakdown of the transparent portion of dye layer will occur over a long period of time as a natural process. This process, which has its roots in chemical kinetics, can take several years in normal environment conditions [3]. Higher temperatures and humidity will accelerate this process by increasing the thermal and kinetic energies of the dye molecules.

It is well known that temperature and humidity are among the most important factors affecting the life expectancy of optical discs. Yet, there is another important factor that has not been so well investigated. Light exposure can increase the rate of dye degradation precisely because the organic dye used in recordable media is light sensitive. This study also addresses this issue.

The effect of these processes can be modeled using various techniques including the Eyring model [4], which is derived from the study of chemical kinetics. The Eyring equation can model the effect of two stresses, such as temperature and relative humidity, on the rate of a reaction or degradation, which can be related to the time-to-failure of the optical disc.

The end of life of a disc can be defined as the time when an uncorrectable error occurs. Although the disc may still be readable after this point, some information has been lost. Consequently the life expectancy of a disc is the period of time in which the information recorded on the disc can be retrieved without loss. In an ideal case, the real time taken for actual failure to occur would be measured and used as the time to failure. However, this measurement is impractical to explore the degradation process, since a single end point cannot describe the complex process that led to failure. Instead we use the maximum value of some error rate monitor, whose gradual change can serve as an indicator of the media stability. In this study, the block error rate (BLER) [5] is used to monitor CDs and the parity inner (PI) [6] error rate, as summed over eight consecutive error correction blocks (PIE Sum8) [6], is used to monitor DVDs. A high BLER rate indicates a potential onset of uncorrectable errors (E32) [5] in CDs, and likewise a high PI error rate indicates a potential onset of uncorrectable errors (PO) [6] in DVDs. In both cases, these error rate monitors are used to characterize the extent of media deterioration.

## 2. Experimental Equipment and Procedural Overview

All testing occurred at the National Institute of Standards and Technology (NIST) as part of the digital data preservation program ongoing in the Information Access Division (IAD). Two types of environmental chambers were used for artificially aging the media. Both chambers were designed to allow aging of the media under a controlled environmental condition.

### Temperature and humidity

A Blue M (model: FRM-256B)^1^ environmental chamber was used to control the temperature and relative humidity through various settings of temperature (−18 °C to −93 °C) and relative humidity (5 % to 98 %). The specified control accuracy is ±0.5 °C for temperature and ±1 % for relative humidity (RH) respectively. The test stresses of aging used are given in Table 1.

**Fig. 1:**
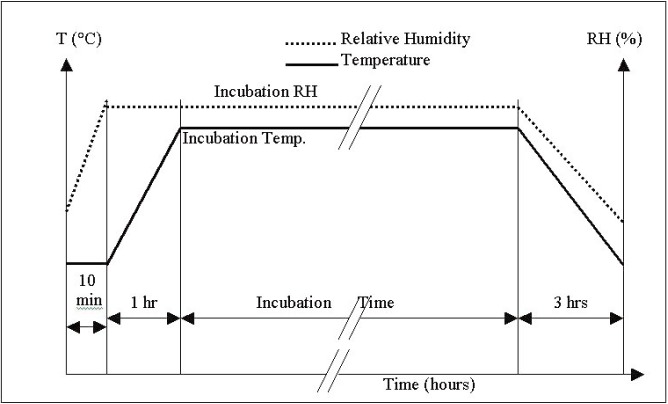
Temperature and RH incubation cycle.

A complete incubation cycle for temperature and RH accelerated testing is shown in Fig. 1. Once at the stress condition, the temperature and RH were held constant for a period of approximately 45 h followed by a gradual return to ambient conditions. Discs were analyzed after each incubation cycle. This cycle was repeated under the same stress condition until the error rate of most discs in the group increased to exceed an upper limit of the error rates (as indicated in the DVD and CD specifications) or until the disc became unreadable.

### Light exposure

**Fig. 2:**
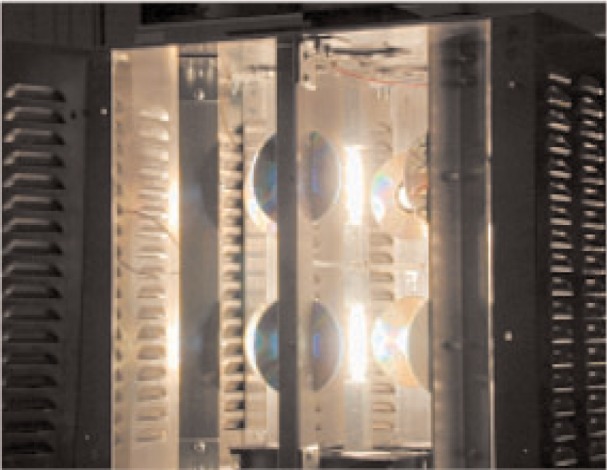
Light Chamber.

A light chamber was designed and built at NIST to meet the requirements for controlled light exposure (Fig. 2). Two cylindrical light bulbs were placed vertically in the center of the chamber, with up to twelve discs placed at equal distance from the light source. Intensity was measured at each disc location to check uniformity. The discs were installed with the recordable side facing the light source.

Two 150 W metal halide (M-H) [7] bulbs were used for the light source, giving a 47.5 mW/cm^2^ light intensity at the disc surface. Light intensities were measured using a Scientech Victor S310 thermo-power meter with shield tube. The wavelength range of the metal halide lamps is similar to sunlight, centered at 500 nm, and partly extending to UV region.

#### Disc Analyzers

In order to monitor the change in the error rate during aging, discs were analyzed after each incubation cycle using disc analyzers. A CD-R analyzer capable of reading BLER (in the case of CD) and a DVD-R analyzer capable of reading PI error was used.

### DVD-R Analyzer

The DVD 1000P analyzer conforms to DVD specifications and was capable of testing electrical, digital, and mechanical parameters in DVDs, including PI errors, PO errors and jitter.

### CD-R Analyzer

The CD CATS SA3 Advanced allowed measurement of all relevant CD disc parameters including BLER, E32 errors and jitter. All measurements are performed according to optical disc industry standards.

#### Test Specimens

Test media were selected randomly from the commercial market. Efforts were made to include all the major dye technologies and many of the main commercial brands. The three dye types typically used in CD-Rs (phthalocyanine, cyanine and azo) were included. The dye types for DVD-R were unknown as no specific information had yet been released. Table 3 shows the CD-R test samples used in this experiment and indicates coating and dye type where possible. Similar information for the DVD-R test specimens was not available. Each sample set had several actual pieces of media to ensure that any particular result was representative of that entire sample.

### 2.1 Key Measured Parameters

#### Jitter

Jitter is the temporal variation or imprecision in a signal compared to an ideal reference clock. It is a measure of how well defined the pits and lands of a disc are. For CD discs, jitter is defined in nanometers (nm), and the CD specification states that jitter should not exceed 35 nm. For DVD recordable discs, jitter is defined in percentage points, and should not exceed 9 %.

#### BLER (CD only)

Block Error Rate is the number of blocks of data that have at least one occurrence of erroneous data. BLER is quantified as the rate of errors (total number of E11, E21, and E31 errors) [5] per second. According to the CD specifications, BLER may be a maximum of 220 per second. Maximum BLER is the maximum BLER measured anywhere on the disc.

#### E32 (CD only)

E32 errors are errors that are uncorrectable by the C2-decoder in the CD error detection and correction system. E32 errors represent lost data and therefore no E32 errors are allowed for in the CD specification.

#### PIE (DVD only)

Data is arranged in DVD discs in a two-dimensional array with appended parity check bits. Each 2-dimensional array is called an error correction code block. Parity Inner errors (PIE) is the number of parity inner rows with errors. According to the DVD specification, any eight consecutive ECC blocks (PI Sum8) may have a maximum of 280 PI errors.

#### POE (DVD only)

Parity Outer errors (POE) are the number of uncorrectable parity outer columns in an ECC block. Since PO errors are uncorrectable by the DVD error detection and correction system, no PO errors are permitted by the DVD specification.

## 3. Results and Discussion

It should be noted that results presented in this paper represent continuous exposure to direct light and extreme temperature/humidity levels. The error rates are not representative of discs stored in typical, normal or ideal storage conditions. The results from these tests are to demonstrate, in terms of error rates, the ability of some DVD and CD media to maintain stability given these extreme conditions.

Also, as stated earlier, each sample set had several pieces of actual media to ensure that results were representative of the entire sample. While there may have been some differences in the results within each sample set, the range was small and thus the results presented here are considered representative of the entire set. Furthermore, particular media from any sample set was subjected to only one particular stress condition.

Results show that the key quality parameters of optical media are altered and error rates increase during exposure to temperature, relative humidity and/or continuous direct light. Since these conditions are key factors in the lifetime of optical media, an estimate for life expectancy can be achieved with a sufficient sample size using various statistical techniques. This investigation, however, was too small to make such an estimate.

**Fig. 3:**
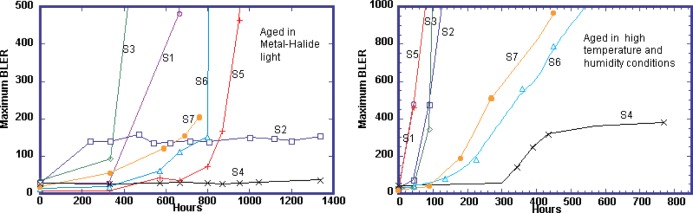
Maximum BLER increase in CD-R when exposed to (A) M-H and (B) extreme temperature/humidity.

The life expectancy of optical media will not be the same for all brands of discs. In a CD-R comparison (see Fig. 3), sample S4, which uses phthalocyanine as the dye and a silver and gold alloy as a reflective layer, is far more stable than any of the other samples during both the temperature/humidity and direct light exposure tests. In a DVD-R comparison (see Fig. 5), sample D2 showed the greatest stability to the temperature/humidity and light exposure tests.

Phthalocyanine based samples S2 and S4 provide very good stability to prolonged direct light exposure as can be seen in the maximum BLER measurements in Fig. 3(A). Both maintain stable BLER levels (following an initial increase in the case of sample S2) beyond 1400 h of exposure to metal halide light whereas other samples have sharp BLER increase within 800 h.

**Fig. 4:**
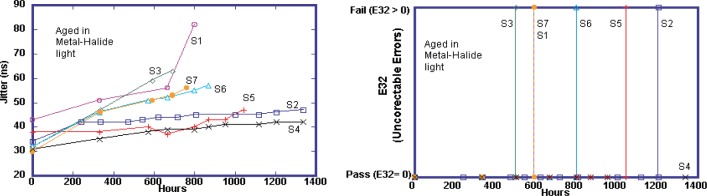
Increase in (A) jitter and (B) E32 in CD-R exposed to M-H light.

Sample S4 also performs the best in temperature and humidity testing. It shows a BLER of less than 400 after 600 h of an extreme temperature and humidity stress condition while all other samples have BLER greater than 600 within 400 h of the same exposure. Some samples (including S1, S2, S3 and S5) show sharp BLER increases within 100 h. Higher stability for sample S4 is also shown for other key measurements including jitter and E32 under all of the accelerated aging stress conditions used (Fig. 4). According to these results, this disc is clearly more suitable for the long-term storage of important digital data.

Sample S2, however, performs poorly in the extreme temperature and humidity testing despite its good stability to direct light exposure. Within 150 h of extreme temperature/humidity aging, a BLER of over 1000 is observed. Sample S2 uses a darkened polycarbonate layer and this seems to have a limiting or filtering effect on the amount of harmful light reaching the data layer.

Samples S1 and S3, both of which use azo dye for the data layer, have higher error rates in both direct light exposure and extreme temperature/humidity testing. Both samples have sharp increases in BLER within 500 h of direct light exposure and within 100 h in extreme temperature/humidity conditions.

Other samples using phthalocyanine, samples S6 and S7, perform well in direct light exposure until approximately 600 h, but then a significant increase in BLER and errors in general is seen. These samples have low errors beyond 100 h of aging in extreme temperature/humidity conditions, but again have sharp BLER increase soon afterwards. Both of these discs have similar stability characteristics, which is not surprising since samples S6 and S7 are from the same manufacturer (although branded differently) and use the same dye and reflective layers.

Sample S5, which uses cyanine for its data layer, performs well under some conditions of direct light exposure but has problems in extreme temperature and humidity conditions. After 600 h of direct light exposure, sample S5 has a BLER of less than 50, second only to sample S4. After 900 h of exposure however, its BLER increases to more than 500. In extreme temperature and humidity testing, sample S5 has an instant and severe increase in BLER.

Comparing the BLER for direct metal halide light exposure from Fig. 3(A) with the jitter and E32 errors from Fig. 4, it can be seen that a high level of correlation exists between the various error indicators of CD-R. It also demonstrates that jitter is a key factor in the quality and stability of CD-R media. As jitter increases, a clear correlation between maximum BLER and jitter emerges. In most results, sudden BLER increases or readability problems occur as jitter increases to approximately 50 nS. Fig. 4(B) also shows that BLER is a good predictor of data loss caused by uncorrectable E32 errors. In the example shown for metal halide light exposure, E32 errors occur for all discs in correlation with a sharp increase of BLER.

Many of the trends observed in the error rates of CD-R are also true for DVD-R. In particular, different samples of DVD-R media show different stabilities during exposure to direct light, temperature and relative humidity. Unfortunately, dye information for DVD-R is less accessible than for CD-R and it is therefore difficult to make a determination of stability based on dye type. However, most DVD-R discs tested are based on a stabilized Cyanine dye. Since results from these samples of similar dye types are quite different, there appears to be varying proprietary modifications made to the dye formulations, and perhaps different manufacturing processes and quality control procedures.

**Fig. 5:**
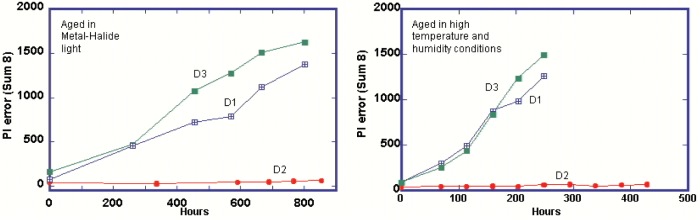
PI (Sum 8) increase in DVD-R when exposed to (A) M-H and (B) extreme temperature/humidity.

Fig. 5 shows that sample D2 is the most stable of the three DVD-R media types tested. PIE for this sample remains low beyond 800 h of exposure to direct metal halide light compared with steady increases in PIE in samples D1 and D3 to approximately 1500 after 800 h.

The stability of sample D2 is further demonstrated when compared to the other samples during exposure to extreme temperature and humidity. Within 200 h, both samples D1 and D3 have reached PIE of approximately 1000 whereas sample D2 remains very low beyond 400 h of exposure.

**Fig. 6:**
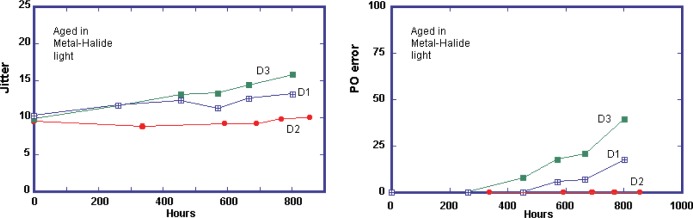
Increase in (A) jitter and (B) POE in DVD-R when exposed to M-H light.

Fig. 6 shows that there is a there is a correlation between the key error rate monitor, PIE, and the onset of uncorrectable parity outer errors (POE), although with the small sample size, it is difficult to identify any clear value of PIE at which POE occurs. And as in the case of CD, jitter appears to have a good correlation with PI errors trends.

## 4. Conclusions

The quantity of media tested in this study was too small to provide the statistical estimation for the life expectancy of the discs but shows relative error trends associated with the different media. A complete study to provide an estimate for the life expectancy is underway at NIST in collaboration with the Library of Congress (LoC).

While there are a number of factors that may contribute to the stability of the CD-R and DVD-R media, dye type is generally considered one of the more important ones. Based on the test results for CD-R media, this expectation appears to hold true, even with mixed results for the dye types. Samples containing phthalocyanine performed better than other dye types. In particular, phthalocyanine combined with a gold-silver alloy as a reflective layer was consistently more stable than all other types of CD-R media. Discs using azo dye as the data layer had less stability in light exposure and temperature/humidity stress testing. Media using cyanine dye performed well when exposed to light but had problems when under temperature/humidity stress conditions.

Although information is less accessible regarding the dye type used in DVD recordable media, it is believed that DVD-R media use a modified form of stabilized Cyanine dye for the recording layer. It is therefore difficult to make any determinations from these results based on dye types for DVD-R media. Furthermore, manufacturers make modifications to the dye to improve its stability or to make it less expensive. This process may result in similar dye types having considerably different qualities, which is shown to be the case in the DVD-R discs tested. And again, as in the case of the CD recordable media, the variation of stability among different brands of DVD recordable media is considerable.

Our results show that the effects of direct light exposure cannot be ignored. The spectral wavelength of metal halide is close to what may be expected within the higher spectrum of sunlight. Depending on the media type and intensity of the light, a disc may fail due to exposure to direct sunlight in as little as a few weeks. This will be especially true when coupled with the heating effect of exposure to sunlight or combined with any other heat source. For archival purposes, however, light is a less challenging issue since it is relatively simple to avoid direct light exposure or prolonged exposure to any damaging light source.

There are a number of physical disc parameters that will provide a good indication of the quality of the media. Based on these results, jitter is a key indicator of media quality in both CD and DVD recordable media. A dye’s ability to maintain well-defined marks is crucial in maintaining low error rates. This also indicates that the dye layer is probably the most significant layer for media stability. Other layers, such as the polycarbonate layer, may also degrade but at a slower rate than the dye layer. Furthermore, a disc with a faded or damaged polycarbonate layer may still have all the data intact and therefore the data may be recovered and migrated to new media. If, however, the dye layer becomes damaged or has degraded, causing uncorrectable errors to occur, the uncorrectable data cannot be recovered. Uncorrectable data error may cause negligible, minimal, or up to catastrophic failure, depending on either the extent or the location of that uncorrectable error within the DVD data structure.

It is demonstrated here that CD-R and DVD-R media can be very stable (sample S4 for CD-R and sample D2 for DVD-R). Results suggest that these media types will ensure data is available for several tens of years and therefore may be suitable for archival uses. Unfortunately, it is very difficult for customers to identify these more stable media.

It is clear that an archive quality grade for media is necessary and should be based on a number of quality parameters rather than brand name or manufacturer. NIST has been leading this effort in consultation with other government agencies and has assisted in the formation of the “Government Information Preservation Working Group” to address this issue. This working group plans to clearly state their needs in regard to the longevity of optical media and work with the optical disc industry to develop requirements for an archival CD or DVD recordable media. A comprehensive study is underway in a collaboration between NIST and the Library of Congress (LoC) with two principle objectives: 1) to determine the life expectancy of DVD recordable media and 2) to develop a test which media manufacturers can use to assign an archive quality grade to their product.

## Figures and Tables

**Table 1:** Temperature and relative humidity stresses

Test stress	Incubation cycle duration	Minimum total time (multiple incubation cycles)
60 °C to 90 °C, 70 % to 90 % RH (various combinations)	Approximately 48 h including ramping	450 h to 850 h (approximately)

**Table 2:** Light exposure stress conditions

Test stress	Incubation period duration	Minimum total time (multiple incubation periods)
Metal Halide	100 h (at controlled temperature)	1400 h (approximately)

**Table 3:** The CD-R specimens for light exposure test

Sample	Coating and Dye
S1	Unknown, Super Azo
S2	Unknown, Phthalocyanine
S3	Unknown, Super Azo
S4	Silver + Gold, Phthalocyanine
S5	Silver, Metal stabilized cyanine
S6	Silver, Phthalocyanine
S7	Silver, Phthalocyanine

## Footnotes

1 Certain commercial equipment, instruments, or materials are identified in this paper to foster understanding. Such identification does not imply recommendation or endorsement by the National Institute of Standards and Technology, nor does it imply that the materials or equipment identified are necessarily the best available for the purpose.
